# Closing a chapter? A protocol for a longitudinal mixed methods study on retirement from elite sport

**DOI:** 10.1186/s40359-023-01422-w

**Published:** 2023-11-07

**Authors:** Noora J. Ronkainen, Michael J. Schmid, Helena Hlasová, Merlin Örencik, Jürg Schmid, Achim Conzelmann

**Affiliations:** Institute of Sport Science, University of Bern, Bremgartenstrasse 145, Bern, 3012 Switzerland

**Keywords:** Athletic retirement, Career transition, Elite athletes, Career termination, Identity, Meaning

## Abstract

Retirement from elite sport represents a major life transition for athletes and requires them to redefine their central life projects, identities, and perhaps even sources of meaning in life. Although an extensive body of literature has identified risk and protective factors in career termination, little is known about the more subjective processes and individual pathways of athletes as they establish their new relationship with work-life and sport. The planned longitudinal mixed methods study follows Swiss elite athletes’ transition with a focus on understanding (1) the relationship between psychological resources, life situations at the end of the sports career, and the retirement process; (2) how athletes’ post-retirement vocational careers interact with their subjective careers and sense of meaningful work; (3) how athletes reconstruct their identities and relationship with sport over time; and (4) how gender shapes athletes’ pathways and reorientation of their life design.

Using a person-oriented approach combined with narrative inquiry, we expect to identify specific types and stories which demonstrate individual differences in career and personal development throughout the transitional period, an understanding of which can be targeted towards support programmes for retiring elite athletes. As the study centralises dimensions of positive psychological functioning (meaning and purpose in life/sport/work, resilience, life satisfaction), it complements previous studies focused on psychological distress and provides much needed knowledge that can be used to foster well-being in athletic retirement. Collaborating with the Swiss Olympic Association helps to ensure that the research findings will be disseminated to relevant end-users and used towards developing socially sustainable elite sport for the future generations.

**Supplementary Information:**

The online version contains supplementary material available at 10.1186/s40359-023-01422-w.

Elite athletes represent a select group of professionals, whose careers, along with the achievements and struggles thereof, are highly visible in the public domain. In Switzerland, where this study is conducted, it has been estimated that 75% of the public follow sports in the media [1]. “The global sporting arms race” [2] and increasing professionalisation of sport has increased the pressure on athletes to push to their physical and mental limits from an early age, and concerns have been raised about the potential consequences on health and well-being lasting long after career termination [3]. While many sport organisations provide support systems for athletes (e.g., dual career and lifestyle management), they typically lose these resources when they leave the elite sport system and need to reconfigure their central life projects. The potentially challenging nature of career termination is illustrated by Fabian Cancellara, multiple world champion and double Olympic gold medal cyclist, who stated that “Quitting is harder than winning races” (translated from German: “Aufhören ist schwieriger als Rennen zu gewinnen”) [4].

Different studies have provided various estimations of how many athletes experience adaptation difficulties. In reviewing recent studies, Cosh et al. [5] reported that somewhere between 18 and 39% of retired athletes experienced mental health challenges (anxiety and depression symptoms) after career termination. Researchers have identified several risk and protective factors that might help to explain different outcomes for athletes, for example, demographical issues (e.g., social status, type of sport, gender, age), athletic identity, voluntary vs. involuntary retirement, athletic achievements, and educational status (for a review, see [6]). However, despite the current conceptualisation of athletic retirement as a transition rather than a single event, few studies have been able to longitudinally follow athletes through the transition to understand how athletes adjust their life narratives and how the different individual and contextual factors interact in individual athletes’ lives to produce these outcomes. Furthermore, little is known about what supports positive indices of well-being in this transition, revealing a remarkable gap in the literature [7].

This study takes its starting point from observations of the potentially challenging nature of the athletes’ retirement transition while bringing new insights into how athletes impose meaning on their retirement transition, how they (re)negotiate purpose in their life projects, and how they make sense of continuity and change in their identities. Using a longitudinal mixed method design (e.g., [8]) which has not been previously used in athletic retirement studies, our team will trace athletes’ pathways from one-year to four-years pre-retirement to one-year post-retirement. This allows for an increased understanding of the different types of journeys and the reorientation processes that athletes go through as they redefine their identities and connections to sport and begin a new chapter of hopefully purposeful projects and careers.

## Athletic retirement: current state of art

Stambulova et al. [9] suggested that the study of athletes’ careers has gone through three distinct stages. In the initiation stage (1960s–1980s), studies focused on athletic retirement and used mostly non-sport concepts and frameworks; in the development stage (1990s), sport-specific frameworks were developed and studies expanded the scope to the whole career; and finally, the present stage (2000s–present) is characterised by the establishment of ”a whole person” and ”whole environment” perspective, including the focus on culturally competent research and applied practice. The retirement research landscape has broadened over time, and contemporary scholarship continues to identifya plethora of variables associated with career transition outcomes (e.g., [6, 7]). This study is specifically informed by the findings pertaining to the effect of identity, career planning and life situation on retirement and adaption. Numerous studies have focused on identity-related questions and reported that high athletic identity and identity foreclosure within sport is negatively associated with quality of transition (e.g., [10, 11]; for an overview, see [6]). However, recent studies demonstrate that athletic identity is not always solely problematic, as it can help athletes sustain a positive relationship with sport [12]. Moreover, Schmid et al. [13] suggested that a strong and exclusive athletic identity might not in itself put athletes at risk; however, these athletes are more likely to make choices (e.g., in education/occupation or social life) that have a harmful influence on the quality of the retirement transition. Furthermore, several studies have demonstrated that engaging in career planning has a positive impact on the quality of retirement transition. The beneficial impact of planning has been shown in relation to financial [14], occupational [15], and psychological [13] considerations for post-sport life.

As evidenced in the literature, the general objective of career termination research has been to determine aspects that influence the quality of transition (for a review, see [6]). What constitutes the quality of transition has been operationalized with different foci, including a psychological (e.g., affective; [11]; self-esteem; [13]), educational and vocational (e.g., [16, 17]) or health (e.g., stress; [18]; well-being; [19]; physical health; [20]) perspective, or a combination of any of these foci (e.g., [21]). However, because different studies have focused on different outcomes the findings are not necessarily generalizable across studies. Furthermore, since researchers’ definitions of career transition success might differ from athletes’ own definitions, it is important to uncover the subjective perceptions of what constitutes a good career and retirement for athletes, how these interact with the objective career paths, and what kind of individual differences exist.

## Areas that need further research

### Athletic career construction in 21st Century

Although athletes’ retirement transition has been studied over several decades, the conceptualisations of the phenomenon and the research questions have changed surprisingly little despite significant changes in elite sport systems and the world of work. As Westerbeek and Hahn [22] noted, elite sport is increasingly undergoing professionalization and commercialization. This could bring additional pressure to athletes, for example, in the form of intensified training and competition schedule, but also more professional opportunities post-retirement (e.g., as a coach or in the sport media). Managing a dual career, social media presence and working with sponsors has become an expected part of contemporary athletes’ pathway, requiring them to be flexible and interesting for the public. The intensive competition and pressure to push to physical and psychological limits makes athletes a vulnerable workforce, and therefore, elite sport is increasingly recognised by researchers as a precarious context [3]. What remains unexplored however, is how these institutional and cultural developments have influenced psychological contracts (i.e., the implicit emotional and practical expectations that athletes might have of the relevant sport organisations and leaders; [23]), career orientations and identity constructions that underpin elite athletes’ trajectories during and after their careers.

While yet to be explored more fully, there are parallels between elite athletes’ careers and the so-called “new careers” studied in vocational psychology. As opposed to the traditional careers involving secure employment and progression in the organisational hierarchy, the new careers demand intensified self-management as well as psychological and physical mobility to manage the increasing number of career transitions [24, 25]. Practising their sport in multiple teams and countries is increasingly common for elite athletes, and cultural transitions are now considered a “quasi-normative” (expected for certain groups of athletes) part of athletes’ careers [9]. As emphasised in vocational psychology, the new careers belong to individuals and not organisations and as career practices have changed, it has been argued that new theoretical concepts are needed to capture the processes of career construction in these individuals.

One of the perspectives responding to the changes in working lives is Career Construction Theory [26] which adopts a narrative perspective on career development and focuses on understanding how individuals produce their career behaviour and adjustment through imposing meaning and direction on their sometimes-fragmented careers. Savickas [26] argued for a greater focus on how individuals draw on their life themes and vocational personality to adjust to career transitions, while remaining faithful to the sense of who they are and maintaining some level of social continuity. Scholarship has moreover emphasised that people adopting new career orientations such as a protean career are typically driven by personal values and are more likely to change jobs or occupations to achieve meaningful work [27]. Therefore, the sense of personal continuity (having sense of direction and continuity in values and meaning structures) has been centralised as a key part of understanding people’s career development and adaptation to career transitions. These perspectives also emphasise that work is only one domain of life-construction, and it needs to fit into the bigger picture of how people aspire to lead their lives. Indeed, meaningful work does not only bring benefits to vocational development, but has been shown to contribute to an overall sense of life meaning [28].

However, these dimensions of career construction have not been studied in the context of athletes’ retirement transition. Therefore, it remains largely unexplored what life themes and work meanings animate athletes’ career construction and how these influence their responses to career termination and orientation to work, sport and life post-retirement. Furthermore, the question remains, which groups of athletes benefit from the current career context in elite sport and can carry the benefits to their post-sport life, and which groups of athletes are disadvantaged and should be targeted in career interventions.

### Retirement transition and relationship to sport

Even though sport is an intensely physical occupation, athletic retirement research has been surprisingly disembodied and most attention has been placed on cognitive constructs such as career planning and coping mechanisms. However, some studies have focused on the changing body image and physical self, indicating that the transition requires adjustment on these dimensions. It has been shown that, after retirement from elite sport, athletes experience lower levels of sport competence, physical attractiveness and self-esteem [29]. More recent studies also indicate that former athletes can be at risk of disordered eating and body dissatisfaction [30].

Some studies in the U.S. collegiate sports have shown that being a former athlete does not ensure sufficient health-related physical activity post-retirement [31]. However, Reifsteck et al. [12] showed that retired collegiate athletes who sustained an athletic identity were more likely to participate in recreational sport and exercise. Later on, Reifsteck et al. [32] also showed that some U.S. collegiate athletes experienced the retirement journey as liberating, since it allowed them to explore new possibilities in the physical culture domain which had been previously unavailable in the highly controlled collegiate sport system.

Despite these steps taken to understand athletes’ relationship with sport post-retirement, it has not been explored how the changed relationship with sport might influence former athletes’ overall sense of meaning in life and life satisfaction. Little is also known about the conditions in which athletic identity might support athletes’ adaptive transition *within* the world of sport, and what kind of changes in athletic identity take place for those who sustain active involvement in sport post-retirement. Therefore, perspectives which explore the nuances in identity such as dialogical self theory [33] can be helpful in broadening our understanding of the different and sometimes contradictory identity positions that athletes develop in relation to sport.

### Gender perspectives

Another dimension of athletic retirement research that needs further research is how gender informs athletes’ pathways and psychological processes in the transition process. Gender has often been reported only as a variable without theoretical considerations, and no significant patterns have been identified in previous reviews on athletic retirement [6, 7]. However, recent studies have identified various ways in which gender influences the pathways of athletes [34–36]. Collectively, these studies have shown that female athletes are more often pursuing dual careers and more committed to education and developing a multi-layered self-narrative than men, whereas male athletes more often prioritise sport over other life domains and receive more encouragement from their social environment for doing so than women. This is also consistent with findings by Schmid, Örencik et al. [16] and López de Subijana et al. [37] that female athletes achieved a significantly higher school-leaving certificate than male athletes. At the mastery level, men are overrepresented in high-income professional athletes [36] and derive a greater income from sponsorship contracts and other sport-related contexts than women [21]. In addition, female athletes on average retire earlier from elite sports and have a shorter athletic career than male athletes [38]. The average duration of sports careers has increased in recent years for these two genders, but more so for male athletes [16]. Concerning the reasons for retirement, family-related reasons seem to be more frequent among women than men [39].

In a narrative study, Ronkainen et al. [40] found that gender scripts were activated in family expectations, ambitions in education, and coach-athlete relationships, pushing female athletes to (consider) retirement earlier than their male counterparts. Similarly using narrative methods, Ekengren et al.’s [34] study pointed out that female athletes were better prepared for retirement than male athletes because they had invested more in education and had developed a more multi-sided self-narrative. These findings indicate that gender plays an important role in understanding the retirement transition, and it is important to use approaches such as narrative inquiry that are sensitive to how psychological processes are culturally shaped. Furthermore, as we argue in the next section, it is important to move beyond identifying general differences in men’s and women’s retirement processes and towards identifying diverse patterns *within* various genders to understand the diversity in how people negotiate gendered expectations and styles in producing their career behaviour.

### The need for a person-oriented perspective

The current scholarship adopts a lifespan, holistic perspective on athletic career development and transitions where dynamic interactions of the factors involved is anticipated [9]. From this perspective, athletic retirement is not the mere sum of each factor contributing to the quality of transition but the consequence of complex person-environment interactions. Therefore, the assessment of the multidimensionality of athletic retirement calls for an adjustment of methods to validly measure athletes’ holistic transitional processes [13]. However, the majority of quantitative studies (for an overview, see [6]) on athletic retirement adhere to a variable-oriented approach based typically on the general linear model establishing a “the more the merrier” relationship between one or more independent and dependent variable/s. This approach entails two major limitations: First, it does not account for the complexity of human development from a dynamic-interactionist perspective [41]. Second, it does not adequately account for reciprocal interactions between variables within an individual [42].

For an individual, the interactions between variables and their subjectively attributed importance are highly specific. Therefore, the dynamic-interactionist perspective recommends observing the process of human development as a whole, rather than considering specific factors individually. This is characterised by its emphasis on the complex interplay of potential development factors and shifts the perspective from a more variable-oriented to a person-oriented perspective [43].

Using a person-oriented approach enables observing non-linear interactions between single characteristics within each individual and therefore can help to identify previously unobserved phenomena [43]. For example, Schmid et al. [44] followed a person-oriented approach to link the resources at the end of an athletic career to the subsequent vocational career. In doing so, they uncovered a more nuanced picture of the different career paths of former Olympic athletes compared to that which a variable-oriented approach could have provided. For example, although the retired Olympic athletes were able to launch a successful vocational career, specific problematic career patterns were found.

Therefore, adopting a person-oriented approach in the current project is particularly useful to account for the heterogeneity within the population with respect to, the dynamic nature of the observed phenomenon and distinct developmental trajectories. In the planned study, we anticipate that the person-oriented approach will be especially valuable for analysing gender- and identity-related questions that have typically been addressed with a variable-oriented perspective, which neglects individual differences in relation to these two constructs.

## Research objectives and questions

The reviewed body of literature provides a platform for the planned study which has the potential to address significant gaps and extend understandings of the diverse pathways through athletic retirement. Using a mixed methods longitudinal design, the current study aims to address the following main research questions:

1. What is the relationship between psychological resources, life situations at the end of the sport career, and the athletic retirement process?
2. How do athletes’ post-retirement vocational pathways (“objective careers”) interact with their subjective sense of career success and meaningful work communicated in their stories?
3. How do sport participation and athletic identity profiles evolve after athletic retirement, and how are these related to athletes’ stories surrounding identity and life meaning?
4. How does gender influence athletes’ adaptation to athletic retirement and reorientation of their life design over time?

## Methods

### Participants

The population of this study consists of all retiring Swiss elite athletes between 2022 and 2024 who are members of the national team in their sport. At this time, the exact sample size is difficult to predict as it is unclear how many athletes will announce their retirement from the sport during the planned period. It has been suggested that up to 7% of athletes in the UK terminate their career annually [45] and it is reasonable to assume similar percentages in Switzerland. Measures of the existing data (see below) indicate that more than  35% within our sample of athletes have already considered athletic retirement and approximately12.5% are planning to retire in the specified period of data collection. Additionally, the Summer Olympics (2024 in Paris) fall in this period, after which an increase in the retirement rate is expected. Overall, we estimate a sample size of around 100 athletes.

Participants for the qualitative interviews are selected as a subset from the quantitative data collection. At the end of the questionnaire, athletes will be asked if they are willing to participate in interviews. Our selection criteria for interviews include full completion of the questionnaire and diversity in sport types and genders. More importantly, however, participants for interviews are selected based on the clusters identified in the quantitative data, with the aim of having interviewees representative of different types. The literature surrounding sample size in qualitative research suggests that sufficient information power is relative to (a) the goal of the study, (b) sample specificity, (c) established theory employed, (d) quality of dialogue, and (e) the analysis strategy [46]. Since we plan to conduct cross-case analysis, seek to answer several research questions, and require data representing different types identified in the quantitative phase, we aim for a relatively large sample of approximately 30 interviews, with balanced gender representation.

### Data gathering and measures

A longitudinal mixed-method design is used to collect the data. The following points of measurement will be used: (1) during the career (see existing data), (2) at athletic retirement (Time 1), and (3) one year after retirement (Time 2).

#### Existing data

The active career development of elite athletes has significant influence on career termination [6], and therefore having knowledge of the active career is necessary for the planned study. This is achieved by drawing on an existing dataset entitled “Life Situations of Elite Athletes” which aims to assess and monitor the life situation (e.g., [36]) and well-being of elite Swiss athletes during their active career. This study entails an annual 20-minute digital questionnaire addressed to all national team athletes in Switzerland and is conducted in collaboration with the Swiss Olympic Association and the Swiss Sport Aid Foundation.

Data has been collected at four measurement points between 2020 and 2023 (in roughly equal intervals). The questionnaires assess the individual life situation from a holistic perspective and include the following aspects: (1) demographic data, (2) the athletic career, (3) the external environment (i.e., occupational career, financial situation, social situation, and support services), and (4) psychological and health-related characteristics (see Table 1). Because of potential adjustments to the COVID-19 pandemic, specific measures have been constructed to assess the consequences of the pandemic on each domain of an athlete’s life.

**Table 1:** Measures of the “life situation of elite athletes” project

Area	Measures
Demographics	Age, gender, sport
Athletic Career^a^	Development of performance: training and competition history Plans of athletic career termination and satisfaction with athletic career
Occupational Career^a^	Educational diploma and job title Time dedicated to education and vocation Satisfaction with occupational career
Financial Situation^a^	Sources of and gross annual income Satisfaction with financial situation
Social Situation	Marital and family status Accommodation status Satisfaction with social situation
Support Services^a^	Occupational and financial support Health and psychological support Satisfaction with support services
Psychological and Well-being	Athlete identity;^b^ self-esteem;^c^ life satisfaction;^d^ resilience;^e^ meaning and purpose;^f^ social support;^g^ perceived stress;^h^ coping mechanisms;^i^ physical and mental health^j^

^a^ Indicates factors for which the impact of COVID-19 was also measured.^b^ Athletic Identity Measurement Scale (AIMS; [47]). ^c^ Rosenberg Self-Esteem Scale (RSES; [48]). ^d^ Satisfaction with Life Scale (SWLS; [49]). ^e^ Brief Resilience Scale (BRS; [50]). ^f^ Meaning and Purpose Scales (MAPS; [51]; and PWB; [52]). ^g^ Multidimensional Scale of Perceived Social Support (MSPSS; [53]). ^h^ Perceived Stress Scale (PSS; [54]). ^i^ Brief COPE [55]. ^j^ WHOQOL-BREF [56].

### New data

Athletes announce their decision to retire to their sports federation, who in turn informs our project partner, the Swiss Olympic Association. The Swiss Olympic Association manages administrative aspects of the retirement and sends out a farewell letter, in which the current project is introduced. Afterwards, the Swiss Olympic Association notifies us and provides the contact information of the retired athletes. Additionally, we are manually searching for retirement announcements through various channels (e.g., newspapers, social media). As soon as we receive confirmation from The Swiss Olympic Association , an email is sent to the retiring athlete inviting them to participate in the first time point of the study. Before starting with the survey, athletes are fully informed about the purpose of the study, their rights as participants and are asked to provide their informed consent. They can indicate their willingness to participate in an interview, which is scheduled for a later date.

Time point two for data collection will begin one year after termination of the career of each athlete. This one-year interval is considered an appropriate interval in prospective career termination research and will allows us to observe the impact of career termination longitudinally while giving athletes time to experience the multi-faceted medium-term effects of career termination [10, 18].

The quantitative phase of the study allows us to observe the relationship between psychological resources, life situations at the end of the sport career, and the retirement process (research question 1). To allow for comparisons to the life situation, an adapted version of the measures of the “Life Situation of Elite Athletes” project (see Table 1) is used and is complemented by questions targeting the career termination adapted from Conzelmann et al. [38]. Specifically, causes for career termination and participation in any kind of intervention to support athletic retirement will be assessed post-retirement. Vocational career situation (research question 2), athletic identity and current sport participation (research question 3) are addressed in the questionnaire, and potential gender patterns (research question 4) are identified when post-retirement life situation is assessed.

At each time point, after completing the questionnaire and giving their consent, the selected athletes (see above) receive an invitation to participate in an interview to take place approximately one month after they have responded to the questionnaires. This slight delay allows the researchers to familiarise themselves with athletes’ responses at the quantitative phase. For capturing non-verbal communication and better rapport building, we prefer in-person interviews whenever possible. To gain in-depth and rich data, we aim for the interviews to be long form and last approximately 90 min for both data collection points.

The interview guide is informed by The Life Story Interview [57] and the Career Construction Interview (CCI) [58]. The Life Story Interview was chosen to encourage free-flowing narration that allows for capturing participants’ experiences and changes in their life situation (research question 1). Accessing the entire life story, beginning with an athlete’s early life, initial sport engagement and eventual retirement allows us to trace how athlete’s self-concepts evolve over time and how they make sense of their lives and sport participation (research question 3). Elements of the CCI are integrated to the interview guide to elicit career stories, which can be analysed for understanding how athletes impose direction and make meaning on their lives and vocational careers (research question 2). Gender dimensions (research question 4) often function as unconscious cultural scripts that give shape to individual stories. Participants are asked to discuss in detail their interpersonal relationships and interactions which allows for analysing how gender influences the intersubjective spaces of meaning-making during athletes’ transition journeys.

### Data analysis

#### Quantitative phase

The data analysis will follow a person-oriented approach. Applied to athletic retirement, cluster and profile analyses can be used to identify common developmental patterns. A key task within the person-oriented approach is the selection of the operating factors because these variables should adequately represent a (sub-)system such as the educational and vocational pathway. If the person-oriented approach is new to the research field, variable-oriented methods can be used to delineate the systems under study and find operating factors [43]. In addition to adopting existing factors previously cited as being central for adaptation to career termination, this current study aims to examine additional relevant factors (meaning, purpose, and resilience) that can provide new insights to the career termination process.

The main statistical approach for our person-oriented design will be the LICUR method (Linking of Clusters after removal of a Residual) [43]. After removing multivariate outliers (residues), this method groups participants into clusters based on a set of variables (i.e., operating factors) for each time point. Subsequently, the resulting classifications are linked and the similarity between the patterns at the different time points (structural stability) as well as the individual transitions between the clusters (individual stability) are determined. If the sample size allows, we will use more advanced analysis strategies, such as latent profile analysis [59] and latent transition analysis [60]. For example, this is likely to be the case for analyses of life situations during the athletic career (see existing data).

#### Qualitative phase

The qualitative data analysis will be conducted with data-driven and theory-driven processes. After the interviews, the qualitative posture of indwelling will be employed in which the audio recordings of the interviews are listened to multiple times and initial notes are made to capture unique features and themes of each narrative. After the interviews are transcribed, the dataset is coded using the principles of thematic narrative analysis [61]. At this stage, the focus is on the content of speech (events, experiences and cognitions captured in the telling). Aligned with the narrative perspective, the segments are kept long so as not to fracture the data and lose the connections within the stories. Athletes’ retirement timelines and stories are compared to identify similarities and differences across cases.

The thematic narrative analysis provides a foundation for specific analyses in relation to different research questions examined in the project. For analysing gender patterns in the dataset, a structural narrative analysis [62] which focuses on the internal coherence, plot, and narrative resources used as building blocks of individual stories will be used. Within the different research questions addressed in the project, additional theory-driven analyses are performed on the dataset. Here, Career Construction Theory [63] informs our reading of the subjective career perspective and identification of life themes; conceptualisations of meaningful work and meaning in life [64] inform the analyses of meaningfulness; and Dialogical SelfTheory [33] is used to capture the internal dialogue and different I-positions that athletes take on with work, sport and other identities. In the qualitative data analysis, research team members will act as critical friends to challenge theoretical interpretations of the data. In the representation of the findings, extensive quotes will be used to transparently demonstrate how researchers have arrived at the interpretations of participants’ stories.

## Discussion

### Methodological reflections

Mixed methods research is increasingly valued for its capacity to draw on the strengths of both quantitative and qualitative research methods to produce knowledge on complex psychological phenomena [65]. In our approach, the quantitative phase builds on the key assumptions of the person-oriented approach [41], that every individual functions as a whole (as a system) and in a specific way. To fulfil the requirement of generating generalizable results, the solution is to identify various athlete-transitiontypes. A narrative approach which informs our qualitative work is similarly built on assumptions about the uniqueness of each individual, and identifying patterns or narrative types is a typical strategy for narrative studies. In our mixed methods design, conducting interviews with a subset of members from each type, allows us to explore these patterns in more detail and with more sensitivity to the context (e.g., [66]). For example, Sorkkila et al. [67] used a similar approach to study the development of burnout in adolescent student-athletes. Using growth mixture modelling, they found four typical profiles in their sample (*N* = 391) and a subset of athletes (*n* = 17) was interviewed to gain more knowledge about their demands and resources. With this approach, the researchers gained a more holistic and comprehensive understanding about the development of burnout in student athletes.

Narrative inquiry has become an increasingly valued methodology for studying career development and transitions because it involves a holistic construction of career trajectories and meanings in peoples’ lives [68]. It is also increasingly used to explore career and identity development in sport due to its ability to capture both personal meanings as well as the broader socio-cultural contexts and narrative resources that make the personal stories possible [69, 70]. Narrative inquiry will be particularly useful in complementing the person-oriented quantitative data as it helps to search for explanations for the observed patterns and to capture the meanings and experiences giving shape to the types identified in the quantitative phase. Additionally, it has the advantage of producing reports that are accessible to a broad audience [62].

### Relevance and impact

There is a justified public demand for a socially and ethically sustainable elite sport. This includes scientific engagement to understand not only the risk factors, but also what a sustainable athletic career looks like and how to promote meaningful lives with fulfilling careers during and after athletes’ engagement with high-performance sport. By studying previously unexamined constructs (meaning, purpose, resilience) and by drawing on narrative theories (Career Construction Theory, Dialogical Self Theory) that have not been applied to athletic career termination specifically, we provide a novel lens for understanding the career termination process.

The research findings will be incorporated into teaching modules and disseminated in peer-reviewed journal articles, conferences, and podcasts to ensure that future sport science professionals will benefit from the knowledge generated in the project. Beyond its scientific impact, we will keep regular contact with our practice partner and engage in various dissemination activities targeting the end users (for example, sport federations, coaches, sport psychology practitioners and career counsellors). To provide practical tools for practitioners, we will produce educational material where former athletes share their “New Chapter Stories” which can be used in workshops and individual counselling. The advantage of making potential role models and “exemplary narratives” available to set a precedent for navigating career development and transitions is documented in previous research [71]. The athletes invited to feature in the educational material are matched to represent different profiles identified in the research and the aim is to make active athletes aware of, and interested in, the multiple pathways that can be pursued after athletic retirement.

### Electronic supplementary material

Below is the link to the electronic supplementary material.

Supplementary Material 1

## Data Availability

Not applicable. This manuscript does not contain any data.
