# Supplementary material for: Closing a chapter? A protocol for a longitudinal mixed methods study on retirement from elite sport
Source: BMC Psychol. 2023 Nov 7;11:376. doi: 10.1186/s40359-023-01422-w (PMC10631078; doi:10.1186/s40359-023-01422-w)
Supplement: Supplementary file 1 — Supplementary Material 1 [file 40359_2023_1422_MOESM1_ESM.pdf]

STROBE Statement—checklist of items that should be included in reports of observational studies

	Item No.	Recommendation	Page No.	Relevant text from manuscript
**Title and abstract**	1	(*a*) Indicate the study's design with a commonly used term in the title or the abstract	1	… A Protocol for a Longitudinal Mixed Methods Study on Retirement From Elite Sport
		(*b*) Provide in the abstract an informative and balanced summary of what was done and what was found	2	The planned longitudinal mixed methods study follows Swiss elite athletes' transition …
**Introduction**				
Background/rationale	2	Explain the scientific background and rationale for the investigation being reported	3–4	… few studies have been able to longitudinally follow athletes through the transition to understand how athletes adjust their life narrative and how the different individual and contextual factors interact in individual athletes' lives to produce these outcomes.
Objectives	3	State specific objectives, including any prespecified hypotheses	11–12	Using a mixed methods longitudinal design, the current study aims to address the following main research questions:….
**Methods**				
Study design	4	Present key elements of study design early in the paper	2, 4, 12–13	A longitudinal mixed-method design …
Setting	5	Describe the setting, locations, and relevant dates, including periods of recruitment, exposure, follow-up, and data collection	12, 15	As soon as we have taken note of retired athletes, an email

				invitation to participate in the study is sent out. … Time 2 will begin one year after termination of the career of each athlete.
Participants	6	(*a*) *Cohort study*—Give the eligibility criteria, and the sources and methods of selection of participants. Describe methods of follow-up<br>*Case-control study*—Give the eligibility criteria, and the sources and methods of case ascertainment and control selection. Give the rationale for the choice of cases and controls<br>*Cross-sectional study*—Give the eligibility criteria, and the sources and methods of selection of participants	12–13	The population consists of all retiring Swiss elite athletes between 2022 and 2024 who are members of the national team in their sport. (…)
		(*b*) *Cohort study*—For matched studies, give matching criteria and number of exposed and unexposed<br>*Case-control study*—For matched studies, give matching criteria and the number of controls per case	N/A	
Variables	7	Clearly define all outcomes, exposures, predictors, potential confounders, and effect modifiers. Give diagnostic criteria, if applicable	14-16	
Data sources/ measurement	8*	For each variable of interest, give sources of data and details of methods of assessment (measurement). Describe comparability of assessment methods if there is more than one group	14-16	*Table 1*
Bias	9	Describe any efforts to address potential sources of bias	15, 18	Additionally, we are manually searching for retirement announcements through various channels (e.g., newspapers, social media) to minimise selection bias.
Study size	10	Explain how the study size was arrived at	12	It has been suggested that up to 7% of athletes in the UK terminate their career annually [45]; it is reasonable to assume similar percentages in Switzerland. Measures of the existing data (see below) of

			1300 athletes indicate that 461 (35.5%) have already considered athletic retirement and 184 (14.2%) are planning to retire in the specified period of data collection. Additionally, the Summer Olympics (2024 in Paris) fall in this period, which typically leads to an increase in the retirement rate. Overall, we estimate a sample size of around 100 athletes.

Continued on next page

Quantitative variables	11	Explain how quantitative variables were handled in the analyses. If applicable, describe which groupings were chosen and why	17
Statistical methods	12	(*a*) Describe all statistical methods, including those used to control for confounding	17
		(*b*) Describe any methods used to examine subgroups and interactions	17
		(*c*) Explain how missing data were addressed	N/A
		(*d*) *Cohort study*—If applicable, explain how loss to follow-up was addressed<br>*Case-control study*—If applicable, explain how matching of cases and controls was addressed<br>*Cross-sectional study*—If applicable, describe analytical methods taking account of sampling strategy	N/A
		(*e*) Describe any sensitivity analyses	N/A
**Results**			
Participants	13*	(a) Report numbers of individuals at each stage of study—eg numbers potentially eligible, examined for eligibility, confirmed eligible, included in the study, completing follow-up, and analysed	N/A
		(b) Give reasons for non-participation at each stage	N/A
		(c) Consider use of a flow diagram	N/A
Descriptive data	14*	(a) Give characteristics of study participants (eg demographic, clinical, social) and information on exposures and potential confounders	N/A
		(b) Indicate number of participants with missing data for each variable of interest	N/A
		(c) *Cohort study*—Summarise follow-up time (eg, average and total amount)	N/A
Outcome data	15*	*Cohort study*—Report numbers of outcome events or summary measures over time	N/A
		Case-control study—Report numbers in each exposure category, or summary measures of exposure	N/A
		Cross-sectional study—Report numbers of outcome events or summary measures	N/A
Main results	16	(*a*) Give unadjusted estimates and, if applicable, confounder-adjusted estimates and their precision (eg, 95% confidence interval). Make clear which confounders were adjusted for and why they were included	N/A
		(*b*) Report category boundaries when continuous variables were categorized	N/A
		(*c*) If relevant, consider translating estimates of relative risk into absolute risk for a meaningful time period	N/A

Continued on next page

Other analyses	17	Report other analyses done—eg analyses of subgroups and interactions, and sensitivity analyses	N/A	
**Discussion**				
Key results	18	Summarise key results with reference to study objectives	N/A	
Limitations	19	Discuss limitations of the study, taking into account sources of potential bias or imprecision. Discuss both direction and magnitude of any potential bias	N/A	
Interpretation	20	Give a cautious overall interpretation of results considering objectives, limitations, multiplicity of analyses, results from similar studies, and other relevant evidence	N/A	
Generalisability	21	Discuss the generalisability (external validity) of the study results	N/A	
**Other information**				
Funding	22	Give the source of funding and the role of the funders for the present study and, if applicable, for the original study on which the present article is based	20	This study is funded by the Swiss National Science Foundation (grant number: 212340). The funders have no role in study design, data collection and analysis, decision to publish, or preparation of the manuscript. The Swiss Olympic Association will provide aid in the recruitment of eligible athletes, increasing their motivation to participate, and access to sport federations.

*Give information separately for cases and controls in case-control studies and, if applicable, for exposed and unexposed groups in cohort and cross-sectional studies.

Note: An Explanation and Elaboration article discusses each checklist item and gives methodological background and published examples of transparent reporting. The STROBE checklist is best used in conjunction with this article (freely available on the Web sites of PLoS Medicine at http://www.plosmedicine.org/, Annals of Internal Medicine at http://www.annals.org/, and Epidemiology at http://www.epidem.com/). Information on the STROBE Initiative is available at www.strobe-statement.org.
